# Backbone Assignment of the MALT1 Paracaspase by Solution NMR

**DOI:** 10.1371/journal.pone.0146496

**Published:** 2016-01-20

**Authors:** Sofia Unnerståle, Michal Nowakowski, Vera Baraznenok, Gun Stenberg, Jimmy Lindberg, Maxim Mayzel, Vladislav Orekhov, Tatiana Agback

**Affiliations:** 1 Medivir AB, PO Box 1086, SE-141 22, Huddinge, Sweden; 2 Swedish NMR Centre, University of Gothenburg, PO Box 465, SE-40530, Gothenburg, Sweden; 3 Centre of New Technologies, University of Warsaw, Banacha 2C, 02–097, Warsaw, Poland; University of Saskatchewan, CANADA

## Abstract

Mucosa-associated lymphoid tissue lymphoma translocation protein 1 (MALT1) is a unique paracaspase protein whose protease activity mediates oncogenic NF-κB signalling in activated B cell-like diffuse large B cell lymphomas (ABC-DLBCLs). ABC-DLBCLs are aggressive lymphomas with high resistance to current chemotherapies. Low survival rate among patients emphasizes the urgent need for alternative treatment options. The characterization of the MALT1 will be an essential tool for developing new target-directed drugs against MALT1 dependent disorders. As the first step in the atomic-level NMR studies of the system, here we report, the ^15^N/^13^C/^1^H backbone assignment of the *apo* form of the MALT1 paracaspase region together with the third immunoglobulin-like (Ig3) domain, 44 kDa, by high resolution NMR. In addition, the non-uniform sampling (NUS) based targeted acquisition procedure is evaluated as a mean of decreasing acquisition and analysis time for larger proteins.

## Introduction

Mucosa-associated lymphoid tissue lymphoma translocation protein 1 (MALT1) has a central role in transcription factor NF-κB signalling [1, 2]. NF-κB controls the expression of numerous anti-apoptotic and proliferation-promoting genes and has a key role in B-cell activation. Constitutive MALT1 activity is one characteristic of specific types of B-cell lymphomas [3, 4], rendering MALT1 as a potential drug target for these malignancies.

MALT1 exerts its regulating function by two routes. Upon antigen stimulation MALT1 acts as a scaffold for a protein complex formed with CARMA1 and Bcl10, the CBM complex [5]. The complex mediates the further events that lead to the nuclear translocation and activation of NF-κB. In addition, MALT1 functions as a protease that acts on several proteins involved in the pathway leading to NF-κB activation [6, 7].

MALT1 is the first human paracaspase identified [2]. Differently from caspases, reported MALT1 substrates are all cleaved directly on the C-terminal side of an arginine residue [6, 7]. Another difference is that MALT1 is not cleaved upon activation [8]. Similarly to caspases, MALT1 is dependent on dimerization for catalytic activity [8]. In vivo this is accomplished by ubiquitination of a single lysine residue [9]. In biochemical assays it has been found that high concentration of a kosmotropic salt increases MALT1 catalytic activity [7], probably by promoting dimerization.

Full length MALT1 is a 93 kDa protein consisting of 823 amino acids (Uniprot Q9UDY8). The sequence folds into five domains: the N-terminal DEATH domain, two immunoglobulin-like domains (Ig1 and Ig2), the caspase-like domain (Casp) and a third immunoglobulin-like domain (Ig3) followed by an unstructured C-terminal tail. The structures of individual domains and combinations thereof have been solved by X-ray crystallography [8, 10–12]. In recent studies MALT1 truncated to the caspase-like and Ig3 domains, MALT1_Casp-Ig3_ was used in characterising the structural determinants of activation [8]. The studies revealed that both *apo* MALT1_Casp-Ig3_ (substrate/ligand-free) and MALT1_Casp-Ig3_ in complex with the irreversible substrate mimetic ligand (z-VRPR-mono fluoro-ketone) forms dimers. However, the trigger of proteolytic activity is substrate-induced. Activation involves stabilisation of loop regions in the caspase domain by the ligand, resulting in the alignment of the catalytic machinery and folding of the substrate binding pocket. In absence of ligand, the loops are disordered and the substrate binding pocket is collapsed.

The flexibility of the major loops in the caspase domain and their relationship with the Ig3 domain seems central to MALT1 activation. Solution NMR is the method of choice for studying such flexible parts in proteins. As the first step towards NMR spectroscopy elucidation of the structure and dynamics of MALT1_Casp-Ig3_, in solution,_._ we report, the ^15^N/^13^C/^1^H backbone assignment of MALT1_Casp-Ig3_ in its ligand-free state by high resolution NMR. In addition, we for the first time show that the targeted acquisition (TA) [13–15] procedure, which previously has been used only for intrinsically disordered proteins and small globular proteins, perform just as well also for a relatively large 44 kDa protein.

## Materials and Methods

### MALT1Casp-Ig3(338–719) expression and purification

The human MALT1_Casp-Ig3_(338–719) construct, consisting of the catalytic domain and the Ig3 domain, was chosen for NMR studies. DNA encoding this sequence and a C-terminal His_6_ tag was inserted between the NdeI and XhoI sites in the expression vector pPET21b (Novagen). Expression of the protein was performed in *Escherichia coli* strain BL21 Star (DE3) (Invitrogen) at 37°C and with 50 μg/ml carbenicillin as the selective antibiotic. For isotope labelling, ^15^N/^13^C/^2^H-labeled minimal medium based on M9 was prepared in D_2_O. At an OD_600_ of approximately 1, induction of the protein expression was started by adding IPTG to 0.4 mM. The cultures were incubated at 18°C for an additional 6 hours. The cells were harvested by centrifugation at 6000g for 20 min and subsequently re-suspended and lysed by high pressure (1,7 kbar) in a Cell Disruptor (Constant Systems). The lysate cleared by centrifugation at 48000g.

The protein was subsequently purified by 3 chromatographic steps. First, an Immobilized metal ion affinity chromatography (IMAC) step on a Ni^2+^ Sepharose 6 Fast Flow column (GE Healthcare) was performed. Second, Ion-exchange chromatography (IEX) on a Q-Sepharose HP column (GE Healthcare) was carried out. Third, a size exclusion chromatography (SEC) step using a HiLoad 16/600 Superdex 200 prep grade column (GE Healthcare) was performed. The buffer was subsequently exchanged to a buffer suitable for NMR experiments on Bio-Spin P-6 gel columns (BioRad). Final yield from a culture of 8 litres was approximately 10 mg of purified protein after concentration to 0.5 mM.

### Backbone assignment of MALT1_Casp-Ig3_(338–719)—the conventional approach

The NMR samples contained approximately 0.5 mM ^15^N/^13^C/^2^H-labeled protein in 10 mM Tris pH 7.5, 50 mM NaCl, 1 mM TCEP-d16, 0.002% NaN_3_, 10 μM DSS-d6 and 10% D_2_O. NMR experiments were acquired on a Bruker Avance III spectrometer operating at a frequency of 700 MHz for ^1^H using a 5 mm cryo-enhanced inverse resonance QCI HFCN probe at 298 K. To enable backbone assignment, transverse relaxation optimized spectroscopy (TROSY)[16–18] versions of HNCO[19], HNCA[19], HN(CO)CA[20], HNCACB[20] and HN(CO)CACB[20], using gradient echo-anti echo TROSY and ^2^H-decoupling, were acquired together with a 2D ^1^H-^15^N TROSY. The total acquisition time sums up to 484 hours (about 3 weeks) and relevant parameters are displayed in Table 1. The backbone assignment was performed manually in CcpNmr Analysis 2.2.2[21].

**Table 1:** Parameters used for acquisition of 3D NMR spectra using the conventional approach (CA) and Targeted Acquisition (TA).

Experiment	HNCO		HN(CA)CO		HNCA		HN(CO)CA		HNCACB		HN(CO)CACB	
Approach	CA	TA	CA	TA	CA	TA	CA	TA	CA	TA	CA	TA
**Number of scans**	4	8	-	16	16	8	16	8	64	16	32	16
**Sparse, %**	100	30	100	19	100	19	100	19	100	19	100	19
**Maximum evolution time (ms)**												
**F3 (**^**1**^**H)**	91	92	-	92	91	92	91	92	91	92	91	92
**F1 (**^**13**^**C)**	9	25	-	25	9	12	9	12	7	12	7	12
**F2 (**^**15**^**N)**	11	22	-	22	11	22	11	22	9	22	11	22
**Measurement time (hours)**	22	13	-	17	46	9	46	10	224	44	146	44

The relaxation delay was set to 1 s in all experiments.

### Backbone assignment of MALT1_Casp-Ig3_(338–719) using the Targeted Acquisition (TA) approach

A standard set of 3D TROSY-based triple resonance experiments with deuterium decoupling was acquired using iterative nonuniform sampling (NUS) [14],[22]. The TA procedure was used for automatical processing and analysis of spectra. TA allows a significant reduction in experiment and analysis time for sequential backbone assignment of proteins and has been described in detail previously[13–15]. Essentially, simultaneous co-processing with multi-dimensional decomposition (co-MDD) of all triple-resonance spectra[15] followed by the automated hyper-dimensional spectrum analysis generates clean peak lists with already prebuilt spin systems that can be efficiently used by automated assignment software AutoAssign [23]. The process is repeated (to accumulate more data in the experiments) and iterated until the assignment is sufficiently complete or converges. A 30% sampling schedule was used for the HNCO spectrum and a 19% sampling schedule was used for all other 3D spectra, yielding a total acquisition time of 136 hours (about 1 week). All spectra were acquired at 298 K on a Bruker Avance III HD 800 MHz spectrometer equipped with a 3mm CPTCI cryo-probe. Peak lists generated with the TA procedure were validated by manual analysis of the spectra in SPARKY[24]. Relevant parameters are displayed in Table 1.

### Estimation of the secondary structure of MALT1_Casp-Ig3_(338–719)

To estimate the secondary structure in MALT1_Casp-Ig3_(338–719), random coil chemical shifts corrected for nearest-neighbour effects[25] were subtracted from ^13^C’, ^13^Cα and ^13^Cβ chemical shifts corrected for deuterium isotope shifts[26] and plotted against the amino acid sequence of MALT1_Casp-Ig3_(338–719). The chemical shift index (CSI) for each carbon type was calculated using a cut-off at ± 0.7 and averaged over all three carbon types to a “consensus” CSI. The secondary structure estimated from the chemical shifts was then compared to the secondary structure of the previously reported X-ray structure of *apo* MALT1_Casp-Ig3_[8] (3V55) and of our in house X-ray structure of *apo* MALT1_Casp-Ig3_ (data not published).

## Results and Discussion

### MALT1_Casp-Ig3_(338–719) assignment by NMR

**Fig 1:**
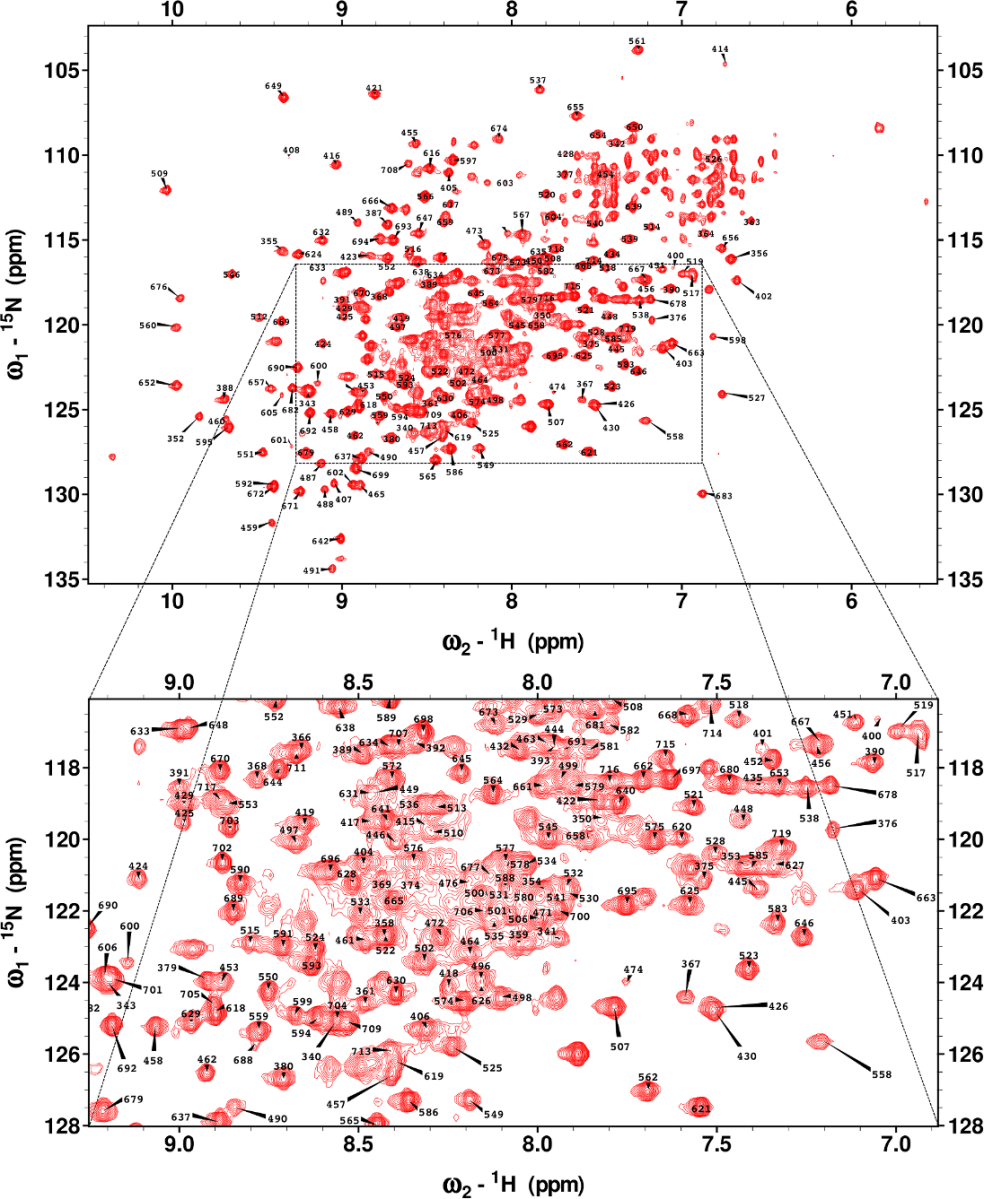
^1^H-^15^N TROSY spectrum of MALT1_Casp-Ig3_(338–719) with the assigned amino acid residue number annotated.

Combining the conventional and TA approaches allowed assignment of 74% of non-proline backbone ^15^N and ^1^H^N^ (Fig 1), 80% of ^13^Cα, 75% of ^13^Cβ and 76% of ^13^CO. The ^1^H, ^13^C and ^15^N backbone chemical shifts were referred to DSS-d6, ^13^C and ^15^N chemical shifts were referred indirectly, and have been deposited in the Biological Magnetic Resonance Data Bank[27] (http://www.bmrb.wisc.edu/) with the BMRB accession code 25674.

### Evaluation of the TA approach

**Fig 2:**
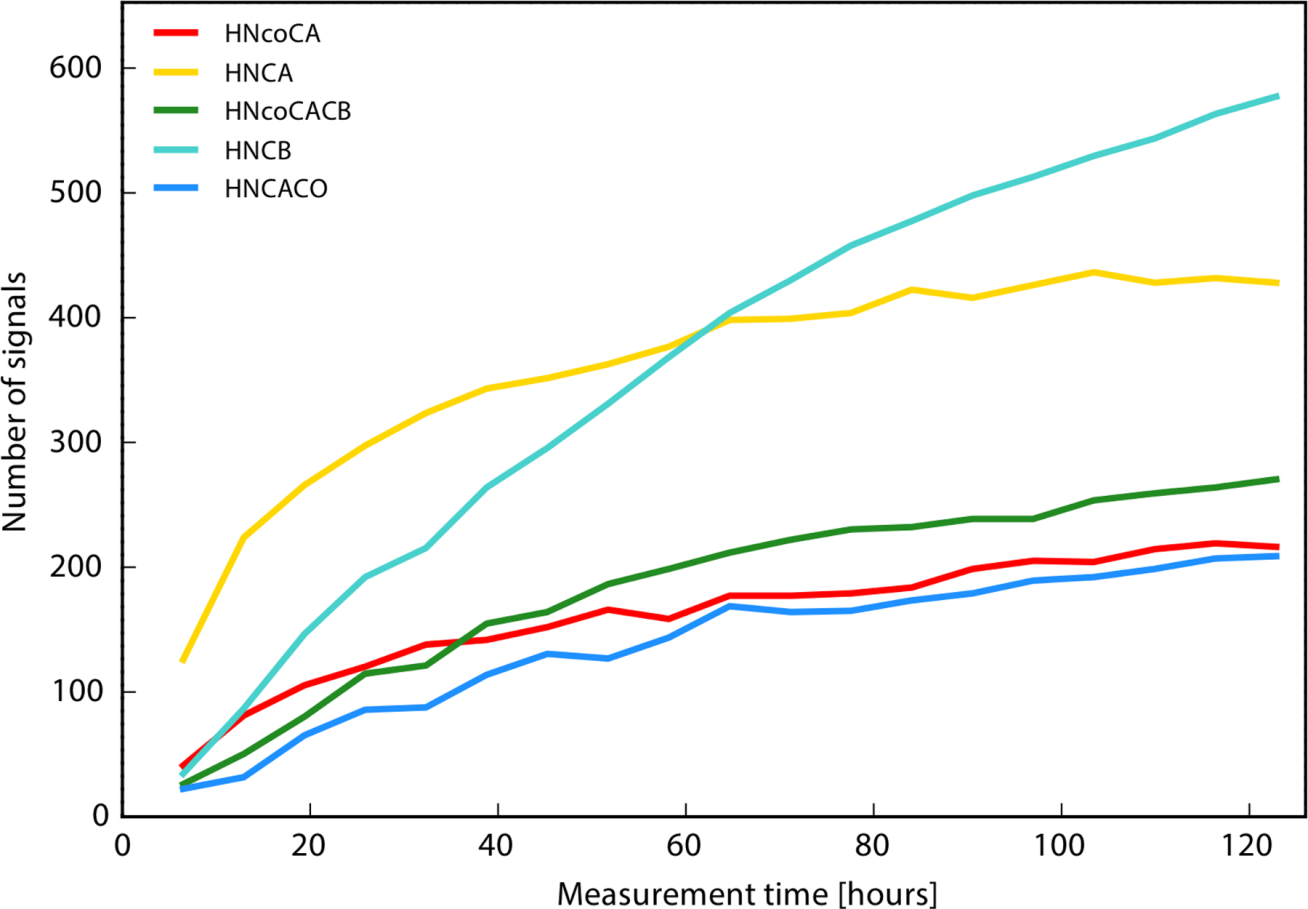
Peak appearance progress during the course of the TA procedure for the MALT1 sample. The horizontal axis shows the total measurement time excluding the HNCO experiment, which was recorded prior to the TA. The spectral processing and analysis was done automatically during the course of the data acquisition.

Apart from obtaining the backbone assignment of MALT1, the secondary goal of this project was to evaluate the performance of the TA approach for a large triple labelled protein. Fig 2 shows the build-up of the peaks with the measurement time, i.e. with increasing number of data points in individual backbone experiments. The peak counts do not level-off at the end of the TA procedure, suggesting that further measurements might improve the result of the assignment. However, the sample stability proved to be the limiting factor. Since the assignment was already obtained using the conventional approach, we decided that the TA data set accumulated during one week was sufficient for the purpose of evaluation of the TA procedure. To make a fair comparison, it should be noted that the TA procedure in this work included modifications that could have benefited the conventional approach as well. These are running the experiment at higher magnetic field (800 MHz), which improves sensitivity and resolution, and use of an additional HN(CA)CO experiment that increases the success and reliability of assignment, albeit in expense of extending the total measurement time.

To evaluate the automated peak picking algorithm used in the TA procedure, all of the spectra were manually carefully analysed in SPARKY[24]. In Table 2 it is clearly seen that the TA approach was successful. Specifically, almost as many peaks were picked when using the TA approach as when using the conventional approach, using only one third of the acquisition time and only one NMR sample instead of two samples. Furthermore, during this measurement time, it was possible to include an additional experiment, the HN(CA)CO, which was essential to disambiguate assignments for several residues and correct a few errors in the assignment produced by the traditional approach.

**Table 2:** Comparison of automated and manual peak picking of the spectra in the TA approach.

	SPECTRA	HNCO	HN(CA)CO	HNCA	HN(CO)CA	HNCACB	HN(CO)CACB
MANUAL	Peaks^a^	358	280	492	268	726	291
AUTOMATIC	Peaks^b^	387	224	459	232	619	290
	False peaks^c^	14	2	2	3	1	3
	Peaks picked twice^d^	15	4	-	-	-	-
	Correct not visible^e^	-	-	2	1	-	2
	New peaks^f^	-	3	3	-	—	5
	Missing^g^	-	65	40	40	108	11

^a^ Peaks picked manually from data acquired by targeted acquisition.

^b^ Peaks picked automatically.

^c^ Peaks picked automatically which were not visible in TA or in the conventional spectra.

^d^ Peaks that were picked twice by the automatic method.

^e^ Peaks picked automatically that were correct (visible in conventional spectra) while not visible in TA spectra.

^f^ Automatically picked peaks that could not be verified.

^g^ Additional peaks that were found in TA spectra upon manual inspection.

It should be noted that peak picking in the HNCO spectrum is semi-automated and its correctness, which is insured by manual verification of the peaks in this spectrum, is crucial for the automated peak detection in all other spectra. Thus, the statistics on the quality of the peak lists refer to all spectra except for the HNCO. The number of false peaks in these spectra is lower than 0.8% in total. For an additional 0.6% of the peaks the obtained data does not allow discrimination between true or false. In addition to the automated peak list, careful manual peak picking allows finding approximately 5% to 20% more peaks and in case of the weakest spectrum, HN(CA)CO, almost 30% more peaks. This was expected, since the TA peak counts did not level-off in our data set (Fig 2).

Comparison of the backbone assignment obtained using the conventional and TA approaches is presented in Table 3. Overall the presented results clearly show that TA can be reliably used for in backbone assignment of large proteins. We clearly show that the method can be used to speed up data acquisition and analysis for backbone assignment of proteins with size of over 40 kDa.

**Table 3:** Summary of the total chemical shifts assigned using the conventional approach and picked (automatically and/or manually) using the TA approach.

	H	N	CO	CA	CB
Total chemical shifts assigned	272	272	289	304	271
Chemical shifts assigned by conventional NMR	264	264	255	283	256
Chemical shifts confirmed by TA	256	256	275	288	239

### The secondary structure of MALT1_Casp-Ig3_(338–719)

**Fig 3:**
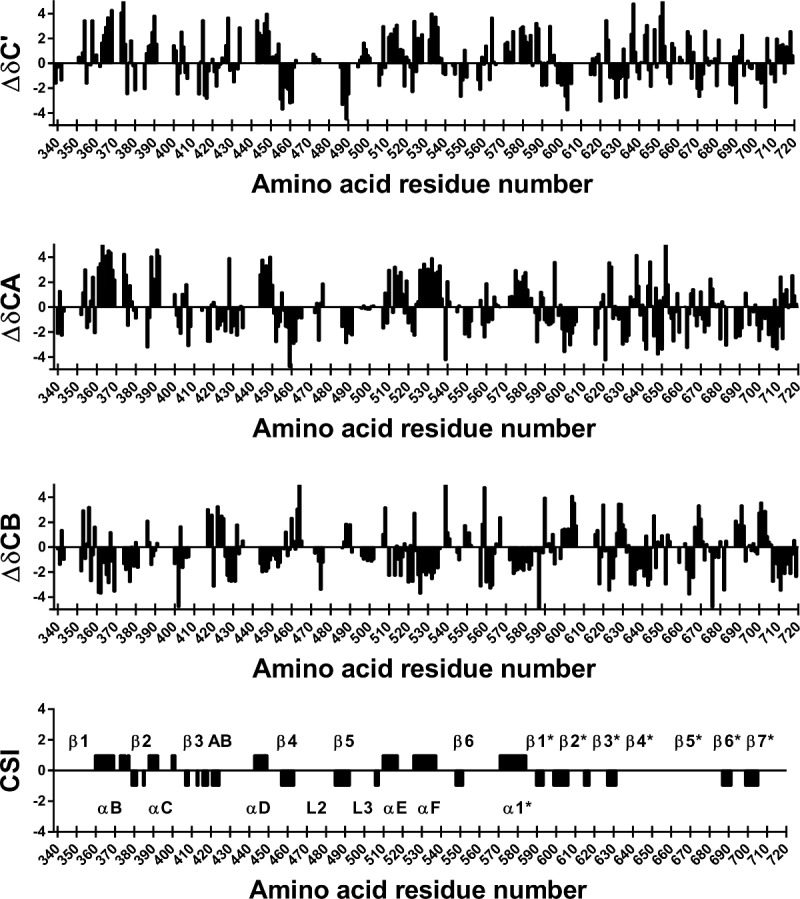
Estimate of the secondary structure in MALT1_Casp-Ig3_(338–719). Secondary chemical shifts (Δδ) were calculated by subtracting random coil chemical shifts corrected for nearest-neighbour effects from ^13^C’, ^13^Cα and ^13^Cβ chemical shifts corrected for deuterium isotope shifts. Consecutive values above 0.7 indicates alpha helix, while consecutive values below -0.7 indicates beta strand for Δδ^13^C’ and Δδ^13^Cα. The opposite is true for Δδ^13^Cβ. The CSI for the three nuclei were averaged and reported as a “consensus” CSI. β3, β3A and β3B are denoted β3 AB in the Fig. The star (*) indicates that the secondary structure is part of the Ig3 domain.

**Fig 4:**
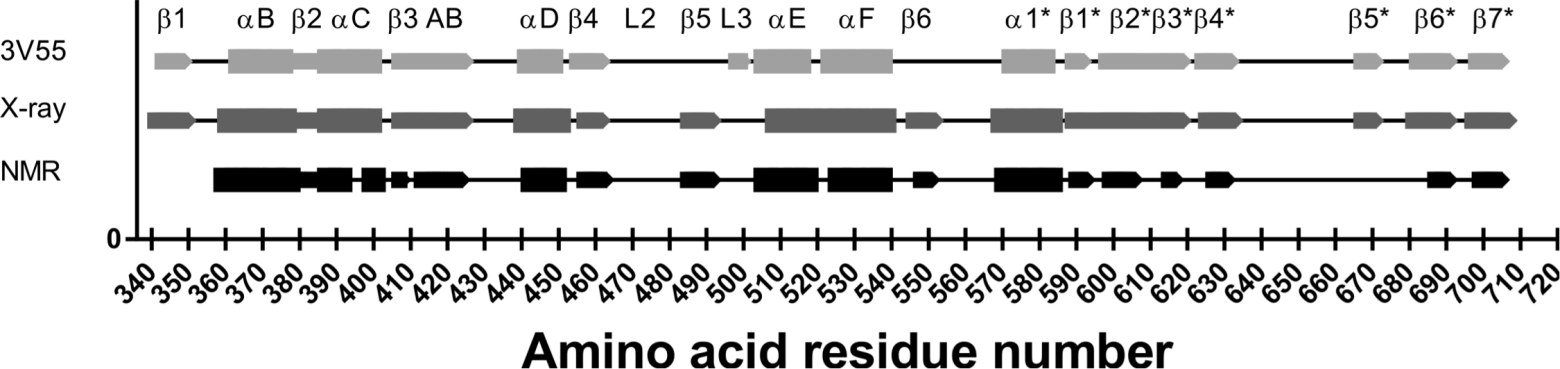
Estimated secondary structure from NMR experiments (in black) compared to secondary structure from the in-house X-ray structure (in dark grey) and from the published X-ray structure of *apo* MALT1_Casp-Ig3,_ PDB ID: 3V55 (in light grey). Alpha helices are indicated with a greater symbol size than the beta-sheets. β3, β3A and β3B are denoted β3 AB in the Fig. The star (*) indicates that the secondary structure is part of the Ig3 domain.

The secondary chemical shifts of ^13^C’, ^13^Cα and ^13^Cβ were calculated and is shown in Fig 3 together with the “consensus” chemical shift index CSI. The CSI derived secondary structure was compared to the secondary structure of the *apo* MALT1_Casp-Ig3_ crystal structures (unpublished in-house structure and published, PDB ID: 3V55), to validate the assignment. As can been seen in Fig 4 the secondary structure estimated from chemical shifts is close to identical to that of the X-ray structures. However some small differences are seen. Most of these differences, i.e. β1, gap in αC, gap in β3AB, gap between β2* and β3* can be explained by gaps in the assignment that creates gaps in the secondary structure estimation. These differences do not imply any real difference between the X-ray structure and the NMR assignment, merely that we do not have enough information to estimate the secondary structure from chemical shifts in these parts of the protein. Further β5* was not identified as a β-strand based on the strict criteria used for the “consensus” CSI. However, if the threshold is lowered slightly also this secondary structure element would be identified. All secondary structure elements except β1 and β5_Ig3_ (denoted β5* in Fig 4) could be assigned based on the data, i.e. αB, β2, αC, β3, β3A, β3B, αD, β4, loop 2 (L2), β5, loop 3 (L3), αE, αF, β6, α1_Ig3_, β1_Ig3_, β2_Ig3_, β3_Ig3_, β4_Ig3_, β6_Ig3_, β7_Ig3_. The different oligomerisation states of apo MALT1_Casp-Ig3_ in solution (monomer) and crystals (dimer) are not reflected by great differences in secondary structure, suggesting that the secondary structure elements necessary for oligomerisation are pre-formed in solution.

## Conclusions

In this work we present the NMR backbone assignment of *apo* MALT1_Casp-Ig3_ in solution (monomer) and show that the secondary structure deduced from the chemical shifts resemble the secondary structure observed in the *apo* X-ray structures (dimer). In addition, we tested the performance of the TA assignment procedure for a large protein system and demonstrated for the first time clear benefit of the approach in reducing measurement and analysis time.

The nature of MALT1 activation and regulation suggests a highly dynamic system in solution. Key to understanding such system is to acquire knowledge on the conformational ensembles and their equilibrium to help elucidate the structure recognised by substrate. For ligand free MALT1_Casp-Ig3_ this includes retrieving structure-dynamics information on the major flexible loops in the caspase domain, and their structural relationship with the C-terminal Ig3 domain. This necessitates solution-based techniques as a complement to X-ray crystallography. The ^15^N/^13^C/^1^H backbone assignment of MALT1_Casp-Ig3_ presented in this report forms a spring board to such future studies aimed at clarifying the role of MALT1 structure-dynamics in substrate recognition and processing.
